# A systematic comparison of in-house prepared transfection reagents in the delivery of mRNA or DNA to a wide range of cultured cells

**DOI:** 10.1016/j.jbc.2025.110742

**Published:** 2025-10-03

**Authors:** Ravi Ojha, Emilia Timin, Leonora Szirovicza, Wujun Xu, Jussi Hepojoki

**Affiliations:** 1 Faculty of Medicine, Medicum, Department of Virology, University of Helsinki, Helsinki, Finland; 2 Faculty of Science, Forestry and Technology, Department of Technical Physics, University of Eastern Finland, Kuopio, Finland; 3 Vetsuisse Faculty, Institute of Veterinary Pathology, University of Zürich, Zürich, Switzerland

**Keywords:** transfection, plasmid, mRNA, cell culture, protein expression

## Abstract

Transfection is a fundamental molecular biology technique, enabling gene editing, protein expression, and vaccine development. However, transfection efficiency and cytotoxicity vary widely between reagent and cell type, necessitating optimization. Commercial reagents such as Lipofectamine 2000 and FuGENE HD are widely used for their high efficiency, but they are expensive and the efficiency can associate with cytotoxicity. In-house alternatives such as linear PEI (25 kDa and 40 kDa) and cationic lipids—1,2-di-O-octadecenyl-3-trimethylammonium propane and 1,2-dioleoyl-3-trimethylammonium-propane—combined with dioleoylphosphatidylethanolamine (DOPE) offer cost-effective options, but their performance across diverse cell types and nucleic acid type (RNA or DNA) remains insufficiently characterized. This motivated us to systematically evaluate transfection efficiency, cytotoxicity, and complex stability of these in-house reagents (DOPE: 1,2-dioleoyl-3-trimethylammonium-propane and DOPE:N-[1-(2,3-dioleyloxy)propyl]-N,N,N-trimethylammonium chloride tested at molar ratios of 0.5:1, 1:1, and 2:1) over a broad range of reagent to nucleic acid ratios, using plasmid DNA and mRNA encoding mCherry. We performed transfections across 14 cell lines derived from human, monkey, frog, snake, and rodent tissues. We utilized automated fluorescence microscopy for quantifying transfection efficiency, luminescence-based viability assays for cytotoxicity, and studied complex stability during storage at 4 °C (0, 4, and 24 h) through transfection. Results revealed cell line-dependent differences in transfection efficiency and showed in-house cationic lipid formulations to have a high mRNA transfection efficiency with low cytotoxicity. Lipofectamine 2000 and PEI 40k formed the most stable DNA complexes, but with higher cytotoxicity. This study provides a comprehensive reference for selecting customizable, cost-effective transfection reagents for specific cell and nucleic acid types.

Transfection is a technique widely utilized in molecular biology and biomedical research for introducing exogenous DNA or RNA into cells (1, 2), to study, for example, cellular processes, viral infections, the molecular mechanisms of diseases, and gene transfer and editing (3). The selection of an optimal delivery (biological, chemical, or physical) method depends on several factors, including the cell type, origin, and the nucleic acids to be introduced. An ideal transfection method should consistently provide high efficiency, minimal cytotoxicity, and maintain the physiological integrity of the transfected cells (3). Here, we focus on chemical transfection methods that were among the first techniques developed for introducing foreign nucleic acids into mammalian cells. The advantages of chemical transfection include relatively low cytotoxicity, absence of mutagenesis, and lack of constraints on the size and type of nucleic acids being delivered (4, 5). Chemicals, such as calcium phosphate, polycationic agents, and dendrimers facilitate nucleic acid transfer through forming complexes that facilitate uptake *via* endocytosis or phagocytosis (4, 5, 6, 7). Furthermore, successful gene expression depends on the type of nucleic acid; DNA must be transported into the nucleus, while RNAs, for example, mRNA will act in the cytoplasm (6, 7).

The emergence of polyamines and cationic lipids as innovative carriers for therapeutic nucleic acids has helped to respond to the growing demand for effective nucleic acid-based therapeutics. Polyamines such as putrescine, spermidine, and spermine are aliphatic amines essential for cell growth, differentiation, and apoptosis (8). At physiological pH, positively charged polyamines interact with negatively charged nucleic acids to form stable polyamine-nucleic acid complexes that enhance cellular uptake and protect nucleic acids from enzymatic degradation (9). Polyamines have been employed in the delivery of siRNAs and plasmid DNA (pDNA) to cancer cells (10), and in mRNA delivery for vaccine purposes (11). However, polyamines and polyamine-based systems can exhibit cytotoxicity at high concentrations (8), due to which optimization is required for balancing the efficacy and safety. Structural modifications of the polyamines or encapsulation within nanoparticles or liposomes can help to improve delivery and decrease cytotoxicity (12, 13) along with codelivery of apoptosis-inhibiting agents (14). PEI is a synthetic polyamide recognized for its high transfection efficiency (15). PEI comes in two forms, linear or branched, and the polymer size contributes to the cationic charge that associates with cytotoxicity; low molecular weight linear PEI is often the choice for *in vitro* applications due to its lower cytotoxicity (16). Linear 25 kDa and 40 kDa forms of PEI offer a balance between transfection efficiency and cytotoxicity, the latter being associated with enhanced binding capacity and transfection efficiency but also with increased cytotoxicity (17). Consequently, researchers frequently optimize PEI concentrations to balance the transfection efficiency with cytotoxic effects (18, 19, 20).

Cationic lipids, another main group of transfection reagents, are amphiphilic molecules characterized by a hydrophilic head group and hydrophobic tails (21). The head group contains the positive charge (is cationic) essential for lipoplex formation through electrostatic interactions with the negatively charged nucleic acids, and in interacting with cell membranes, and in the endosomal release of the lipoplexes (21). Linkers mediate the connection between the cationic head and the hydrophobic tails, and, in addition to aspects like stability, the linker contributes to transfection efficiency by altering the conformational flexibility (21). The tail, that is, the lipid component, allows integration into lipid bilayers, enhancing membrane fusion and stability. Modifications to the head group and tail structure influence the lipid’s physicochemical properties, such as charge density, hydrophobicity, and fluidity, directly impacting their biological functionality (22). Strategies to reduce cytotoxicity include structural modifications to the lipid’s head group and tails, for example, incorporation of hydrophilic moieties can enhance solubility and reduce membrane disruption. Additionally, pH-sensitive lipids that respond to acidic endosomal environments allow targeted release, minimizing off-target effects and improving overall cell viability (23, 24, 25). Transfection utilizing cationic lipids, that is, lipofection was first demonstrated utilizing N-[1-(2,3-dioleyloxy)propyl]-N,N,N-trimethylammonium chloride (DOTMA)(26). The ester analog of DOTMA, 1, 2-dioleoyl-3-trimethylammonium-propane (DOTAP) (27), has also been utilized in nucleic acid delivery since the early days of lipofection. As an example of cationic lipids, DOTAP and DOTMA facilitate electrostatic interactions with nucleic acids, forming lipoplexes that enhance cellular uptake, and following internalization they mediate the release of nucleic acids into the cytoplasm. Helper lipids, such as dioleoylphosphatidylethanolamine (DOPE), are commonly added to improve the stability and fusion properties of the lipoplexes. Additionally, optimizing the lipid composition and formulation parameters, such as charge ratio and particle size, enhances stability and reduces aggregation, ensuring consistent performance (28, 29). While DOTAP and DOTMA exhibit moderate cytotoxicity, optimizing the lipid-to-nucleic acid ratio can mitigate adverse effects and improve transfection outcomes (26, 30, 31). The efficiency is influenced by their formulation and the presence of helper lipids. There are several commercial formulations and reagents available for lipofection, including, for example, Lipofectamine (developed by Invitrogen) or Fugene (developed by Promega) reagents. Lipofectamine 2000 is well-known for its exceptional efficiency in delivering nucleic acids such as pDNA and RNA, into a wide variety of cell types, including both primary cells and hard-to-transfect cell lines (32). Although highly effective within recommended concentration ranges, Lipofectamine 2000 may induce cytotoxic effects at elevated concentrations due to cellular stress and apoptosis (32, 33, 34). Fugene HD also demonstrates high transfection efficiency and notably reduced cytotoxicity profile even at higher concentrations, making it a favorable option for applications that demand high posttransfection cell viability (34, 35).

The molecular mechanisms by which transfection reagents facilitate the delivery of nucleic acids into cells can be broadly categorized into complex formation, cellular uptake, endosomal escape, and nuclear delivery (36). The complex formation between cationic reagents and negatively charged nucleic acids is primarily driven by electrostatic forces (37, 38). Polyamines interact with nucleic acids to create polyplexes (39) and cationic lipids form lipoplexes by encapsulating pDNA or mRNA within lipid bilayers, both protecting the nucleic acids from degradation. The size and charge of these complexes influence cellular uptake, smaller complexes often show higher transfection efficiency due to enhanced interaction with cells. Endocytosis is the prime mechanism of cellular uptake of lipoplexes and polyplexes (15, 40). Once inside the cell, the lipoplexes and polyplexes need to facilitate the release of nucleic acids from endosomes before lysosomal degradation (26). Cationic lipids and polyamines use different mechanisms to promote endosomal escape (39). Polyamines can, for example, induce osmotic swelling by inducing an influx of ions and water, leading to endosomal lysis, thus allowing the escape of nucleic acids into the cytoplasm (15, 39). Cationic lipids facilitate the release of nucleic acids into the cytosol by promoting membrane fusion that can occur at the plasma membrane or in the endosomal route (41, 42). The final step in successful transfection is the delivery of nucleic acids into the nucleus, especially essential for pDNA to exert its biological effects. The nuclear transport of nucleic acids can occur *via* passive diffusion or active transport mechanisms. For pDNA, nuclear pore complexes serve as the entry gateways into the nucleus, with the size and structure of the DNA complex influencing its ability to traverse the nuclear pore complexes. Smaller complexes are more likely to diffuse through the nuclear pore, while larger complexes may require active transport mechanisms (36, 43, 44). For mRNA and other RNA species, the delivery into the nucleus is less critical, as they mainly function in the cytoplasm; however, ensuring the stability and integrity of RNA during the delivery is essential (36).

While transfection formulations such as Lipofectamine 2000 and Fugene HD, as well as their derivatives, provide excellent transfection efficacy and reagent stability, the cost of commercial transfection reagents is a cause of significant consideration for many researchers. Cationic lipids such as DOTAP and DOTMA together with polyamines like PEI provide a cost-effective alternative with relatively good performance and stability (12, 36, 45). The efficacy of these reagents in the delivery of nucleic acids into cultured cells has been covered by a wide range of studies, however, often the studies focused either on DNA or RNA delivery and on a narrow range of cell lines. This motivated us to systematically compare the efficiency of linear PEI 25K, linear PEI 40k, DOTMA:DOPE (at ratios 0.5:1, 1:1, and 2:1), and DOTAP:DOPE (at ratios 0.5:1, 1:1, and 2:1) in the delivery of pDNA and mRNA across a panel of 14 cell lines. We evaluated each reagent at a wide range of concentrations, keeping the nucleic acid concentration constant (*i.e.*, varying reagent to nucleic acid ratios). To facilitate quantification of the transfection efficacy through automated fluorescence microscopy, we used pDNA carrying mCherry open reading frame under CAG promoter and *in vitro* transcribed mCherry mRNA as the transfected nucleic acids. In addition, we studied the cytotoxicity of the transfection reagents at the concentrations found to provide optimal transfection efficacy. To facilitate comparisons, we, in parallel, tested the efficacy of two commonly used commercial reagents, Lipofectamine 2000 (Invitrogen, 11668019) and Fugene HD (Promega, E2311). Rather than providing maximally optimized transfection conditions and percentages of transfected cells, our goal was to identify the ideal reagent or reagents for mRNA and pDNA delivery to each cell line. To reduce the need for optimization in other labs, we further aimed to establish a range of suitable range of working concentrations and cytotoxicity profiles of the reagents tested.

## Results

### Binding of nucleic acids to the transfection reagents

**Figure 1:**
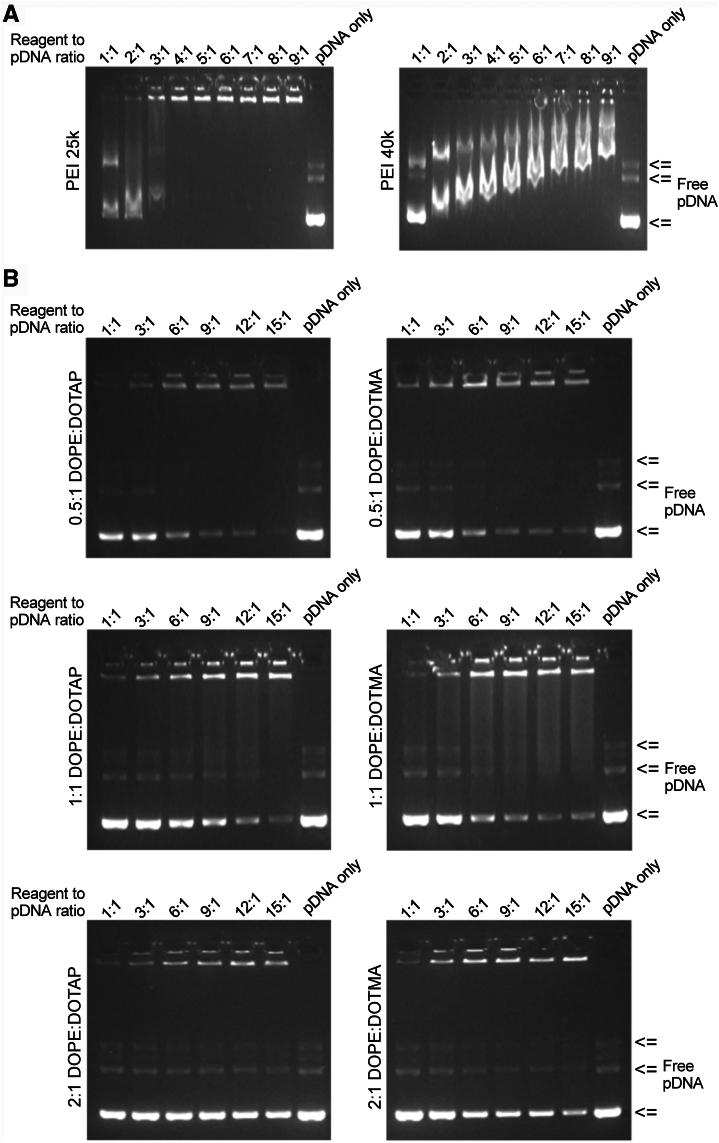
**Complex formation of pDNA with polyamines and cationic lipids as studied by mobility shift on agarose gel electrophoresis.** We mixed a constant amount of pDNA with increasing concentrations of the transfection reagents and separated the resulting complexes on a standard 1% (w/v) agarose gel. *A,* complex formation between pDNA and PEI 25k (*top*) or PEI 40k (*bottom*) at nine different ratios. *B,* complex formation between pDNA and cationic lipids DOTAP and DOTMA in combination with increasing concentrations of helper lipid DOPE, DOPE:DOTAP (0.5:1, 1:1, 2:1), and DOPE:DOTMA (0.5:1, 1:1, 2:1), and at nine different ratios to pDNA. GelRed (Biotium) was used for staining the pDNA, and Gel Doc 2000 (Bio-Rad Laboratories) was used for recording the results. DOPE, dioleoylphosphatidylethanolamine; DOTAP, 1, 2-dioleoyl-3-trimethylammonium-propane; DOTMA, N-[1-(2,3-dioleyloxy)propyl]-N,N,N-trimethylammonium chloride; pDNA, plasmid DNA.

To study the complex formation of the “in-house” transfection reagents, we mixed PEI 25k, PEI 40k, DOPE:DOTAP, or DOPE:DOTMA with pDNA at increasing ratios and used agarose gel electrophoresis to study pDNA mobility as an indicator of complex formation. The results showed PEI 25k to alter the pDNA mobility already at 1:1 ratio, and at 4:1 or higher ratio free pDNA became undetectable, suggesting efficient complex formation (Fig. 1*A*). PEI 40k demonstrated similar, albeit less efficient complex formation from 2:1 ratio onward and demonstrated curious pattern of decreasing mobility at higher PEI 40k concentrations (Fig. 1*A*).

The cationic lipids DOTAP and DOTMA, mixed with the helper lipid DOPE at varying ratios, showed similar complex formation as judged by pDNA gel mobility shift (Fig. 1*B*). For both DOPE:DOTAP and DOPE:DOTMA, the most efficient complex formation occurred at 0.5:1 ratio. The 2:1 ratio showed most amount of free pDNA, suggesting inefficient complex formation or instability of the formed complexes. For the 0.5:1 ratio of both DOPE:DOTAP and DOPE:DOTMA, most of the freely migrating pDNA appeared to be lost at 6:1 to 9:1 ratio. At 1:1 ratio, 9:1 and 12:1 ratios for DOPE:DOTAP or 6:1 and 9:1 ratios for DOPE:DOTMA were needed to produce a similar effect. At 2:1, only DOPE:DOTMA at 15:1 appeared to induce a clear loss of freely migrating pDNA (Fig. 1*B*).

Overall, PEI 25k appeared to be most efficient in forming stable complexes with pDNA at the concentrations applied and analysis conditions used. However, when looking at the pDNA complexes present in the upper parts of the gels, complex formation appeared to take place in practically all conditions tested.

### pDNA and mRNA delivery efficacy of transfection reagents varies between cell lines

After identifying the concentration range for complex formation with pDNA, we proceeded to test the suitability of the reagents in delivering pDNA or mRNA to a panel of 14 cell lines. We employed mCherry encoding pDNA and *in vitro* transcribed mCherry mRNA in these experiments, to enable direct quantification of the results using automated microscopy. In total, we tested ten transfection reagents: Lipofectamine 2000 and Fugene HD as the positive controls, polyamines PEI 25k and PEI 40k, and cationic lipids DOTAP and DOTMA with helper lipid DOPE at ratios of 0.5:1, 1:1, and 2:1. For the positive controls, we examined three reagents to nucleic acid ratios each, 2:1, 4:1, and 6:1 for Lipofectamine 2000 and 1.5:1, 3:1, and 4.5:1 for Fugene HD. For the “in-house” reagents, we assessed a broader range, 1:1, 2:1, 3:1, 4:1, 5:1, 6:1, 7:1, 8:1, and 9:1 for PEI 25k and PEI 40k, and 1:1, 3:1, 6:1, 9:1, 12:1, and 15:1 for both DOPE:DOTAP and DOPE:DOTMA combinations.

**Figure 2:**
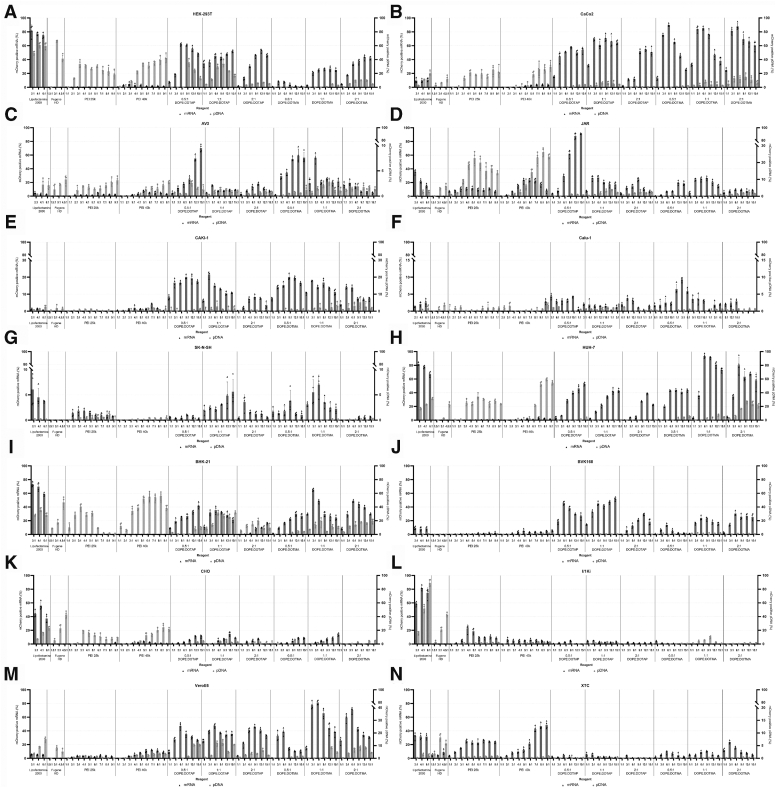
**Comparison of mCherry plasmid DNA and mRNA expression following transfection in different cell lines using various transfection reagents.** The cell lines tested include (*A*) HEK-293T, (*B*) Caco-2, (*C*) AV3, (*D*) JAR, (*E*) Caki-1, (*F*) Calu-1, (*G*) SK-N-SH, (*H*) Huh-7, (*I*) BHK-21, (*J*) BVK-168, (*K*) CHO, (*L*) I/1Ki, (*M*) Vero E6, and (N) XTC. Each cell line was transfected using Lipofectamine 2000, Fugene HD, PEI 25k, PEI 40k, DOPE:DOTAP, and DOPE:DOTMA at varying reagent-to-nucleic acid ratios (*from left to right in each graph*), and the transfection efficiency was quantified through enumerating the cells expressing mCherry using automated fluorescence microscopy. Bar graphs display the mean ± SD from four independent technical replicates, with superimposed scatter plots illustrating the reproducibility of individual replicate measurements. DOPE, dioleoylphosphatidylethanolamine; DOTAP, 1, 2-dioleoyl-3-trimethylammonium-propane; DOTMA, N-[1-(2,3-dioleyloxy)propyl]-N,N,N-trimethylammonium chloride.

In HEK293T cells, Lipofectamine 2000 used at 4:1 or 6:1 reagent:DNA ratio mediated transfection at ∼60% efficiency (Fig. 2*A*). Fugene HD showed similar efficiency when applied at a 3:1 ratio to DNA. PEI 25k showed transfection efficiencies >30% when used at 3:1 or 4:1 ratio to DNA, and a broad range of reagent: DNA ratios showed ∼25 to 30% transfection efficiency. With PEI 40k the transfection efficiency increased with the reagent concentration, and reagent:DNA ratio of 9:1 yielded >40% efficiency. DOPE:DOTAP mixes showed large variation in their transfection efficiencies, DOPE:DOTAP 0.5:1 and 1:1 produced the highest transfection efficiencies (∼35%) at a 9:1 reagent:DNA ratio. For DOPE:DOTAP, the 2:1 mix performed the best with ∼10% transfection efficiency for 6:1 to 12:1 reagent:DNA ratios. For mRNA transfection, Lipofectamine 2000 showed a transfection efficiency of ∼80% for all tested concentrations. Fugene HD could not mediate mRNA transfection, and PEI 25k and PEI 40k showed low transfection efficiencies (∼0.5% and ∼3%, respectively). Among DOPE:DOTAP mixes, the 0.5:1 mix showed the best performance, allowing transfection efficiency of 60% at reagent:mRNA ratios of 6:1 and 12:1. For 1:1 and 1:2 DOPE:DOTAP mixes, several reagent:mRNA ratios produced ∼50% transfection efficiency. For DOPE:DOTMA, the 2:1 mix showed the highest transfection efficiency, reaching almost 50% at a 12:1 reagent:mRNA ratio.

In CaCo-2 cells, Lipofectamine 2000 showed increasing efficiency with higher reagent:DNA ratios, reaching ∼20% efficiency at 6:1 ratio (Fig. 2*B*). The other commercial reagent, Fugene HD, reached ∼5% transfection efficiency at the higher reagent: DNA ratios. PEI 25k demonstrated transfection efficiency of ∼20% over a broad range, 5:1 to 9:1, of reagent:DNA ratios, whereas PEI 40k showed efficiency to increase with reagent:DNA ratio with ∼30% efficiency reached at a 9:1. Of the DOPE:DOTAP mixes, the one with 1:1 DOTAP was the most effective and showed ∼5% transfection efficiency at 9:1 reagent:DNA ratio. With DOPE:DOTMA mixes, 2:1 ratio yielded the highest transfection efficiency (≥10%) over a broad range of reagent:DNA ratios (3:1–12:1). For mRNA transfection, Lipofectamine 2000 showed transfection efficiency increasing with reagent concentration, reaching ∼15% efficiency at 6:1 reagent:mRNA ratio. While Fugene HD and PEI 25k could not mediate mRNA transfection at the concentrations tested, PEI 40k showed transfection efficiency of ∼3.5% at 8:1 to 9:1 reagent:mRNA ratios. DOPE:DOTAP mixed at 0.5:1 or 1:1 showed >70% mRNA transfection efficiency at 3:1 reagent: mRNA ratio, and the 1:1 DOPE:DOTAP mix showed transfection efficiency of >60% of over a broad range of reagent:mRNA ratios. All DOPE:DOTMA mixes showed >80% transfection efficiency when at reagent:mRNA ratios of 3:1 to 6:1, and ∼70 to 80% efficiencies even at 9:1 ratio.

In AV3 cells, Lipofectamine 2000 and Fugene HD yielded transfection efficiencies of ∼3%, both at the highest reagent:DNA ratio applied, 6:1 and 4.5:1, respectively (Fig. 2*C*). Both PEI 25k and PEI 40k showed efficiency similar to the commercial reagents when mixed at 9:1 ratio to DNA. Of the DOPE:DOTAP mixes, 0.5:1 showed the best performance, allowing transfection efficiency of ∼3% when mixed at 9:1 ratio with DNA. Of the DOPE:DOTMA mixes, 1:1 produced the highest transfection efficiency, reaching ∼3% at 9:1 ratio to DNA. For mRNA transfection, Lipofectamine 2000 showed ∼2.5% efficiency at 2:1 ratio to mRNA, the higher reagent concentrations yielded lower efficiency. Fugene HD did not allow mRNA transfection. PEI 25k showed transfection efficiency of ∼4% when used at 5:1 or 6:1 ratio to mRNA. While several PEI 40k concentrations demonstrated similar transfection efficiency, 5:1 ratio to mRNA demonstrated the highest transfection efficiency (∼7%). Of the DOPE:DOTAP mixes, 0.5:1 showed the best performance, reaching ∼70% transfection efficiency when mixed at 15:1 ratio to mRNA. Similarly, the DOPE:DOTMA 0.5:1 mix yielded the best transfection efficiencies among the combinations tested, reaching ∼70% efficiency when mixed at a 9:1 ratio to mRNA.

In JAR cells, Lipofectamine 2000 mediated DNA transfection at <5% efficiency, and the other commercial reagent Fugene HD showed slightly higher efficiency (>5%) over the concentration range tested (Fig. 2*D*). PEI 25k demonstrated transfection efficiency of ∼25% at 5:1 reagent:DNA ratio, and PEI 40k showed similar efficiency at 7:1 to 9:1 ratio. DOPE:DOTAP performed best when mixed at 1:1 ratio; however, the transfection efficiency remained <5% for the tested reagent:DNA ratios. DOPE:DOTMA 2:1 mix showed at best <5% transfection efficiency, and the other DOPE:DOTMA mixes performed at even lower efficiency. For mRNA transfection, Lipofectamine 2000 2:1 reagent:mRNA ratio showed the highest transfection efficiency, ∼35%. Fugene HD could not mediate mRNA transfection. PEI 25k showed >10% transfection efficiency at 4:1 and 5:1 reagent:mRNA ratio, whereas PEI 40k showed >20% efficiency when mixed between 6:1 to 9:1 with mRNA. While DOPE:DOTAP mixed at 1:1 or 2:1 showed transfection efficiencies up to 30%, DOPE:DOTAP 0.5:1 mix showed ≥90% transfection efficiency if mixed with mRNA at 12:1 or 15:1 ratio. Of the DOPE:DOTMA mixes, 1:1 ratio showed best performance, reaching >40% transfection efficiency at 6:1 reagent:mRNA ratio.

In Caki-1 cells, Lipofectamine 2000 allowed transfection efficiency of ∼1 to 2%, and Fugene HD performed at similar level (Fig. 2*E*). PEI 25k and PEI 40k demonstrated slightly higher transfection efficiencies, ∼2% and ∼3%, at respective reagent:DNA ratios of 5:1 and 6:1. Of the DOPE:DOTAP mixes, 1:1 yielded the highest transfection efficiency (∼5%) at 6:1 reagent:DNA ratio. Of the DOPE:DOTMA mixes, 2:1 yielded the highest transfection efficiency, ∼6%, at 9:1 reagent:DNA ratio. For mRNA transfection, Lipofectamine 2000 showed the highest efficiency, ∼1.5%, when used at 2:1 or 4:1 reagent:mRNA ratio. Fugene HD and PEI 25k could not mediate mRNA transfection, and 4:1 reagent:mRNA ratio yielded the highest transfection efficiency (∼2%) with PEI 40k. Of the DOPE:DOTAP mixes, 0.5:1 demonstrated the best overall performance, yielding ∼15 to 20% transfection efficiency at 3:1 to 15:1 reagent:mRNA ratios. The DOPE:DOTAP 1:1 mix showed >20% transfection efficiency at 3:1 ratio to mRNA, however, the other tested ratios showed efficiencies <15%. Of the DOPE:DOTMA mixes, 0.5:1 yielded highest transfection efficiencies (∼20%) when applied at 9:1 or 12:1 ratio to mRNA. The DOPE:DOTMA 1:1 mix showed ∼20% transfection efficiency when used at 3:1 ratio to mRNA.

In Calu-1 cells, Lipofectamine 2000 and Fugene HD demonstrated ∼1% transfection efficiencies at respective reagent:DNA ratios of 6:1 and 4.5:1 (Fig. 2*F*). PEI 25k and PEI 40k showed transfection efficiencies of ∼3% at ratios 9:1 and 7:1 to DNA, respectively. Of the DOPE:DOTAP mixes, 1:1 allowed the highest transfection efficiency, reaching ∼3% when mixed at 12:1 with DNA. The DOPE:DOTMA mixes showed transfection efficiencies reaching ∼1%. For mRNA transfection, Lipofectamine 2000 showed highest efficiency (∼3.5%) when mixed at 6:1 ratio with mRNA. Fugene HD, PEI 25k, and PEI 40k could not mediate mRNA transfection at the tested concentration range. Of the DOPE:DOTAP mixes, 0.5:1 showed ∼3 to 4% transfection efficiency over the tested concentration range, the highest efficiencies recorded at 3:1 and 15:1 ratio to mRNA. While DOPE:DOTAP 1:1 showed lower transfection efficiencies, the 2:1 mix showed ∼4% efficiency when mixed at 9:1 ratio to mRNA. Of the DOPE:DOTMA mixes, 1:1 yielded the highest transfection efficiencies, reaching >9% when mixed at 3:1 ratio to mRNA.

In SK-N-SH cells, the commercial reagents, Lipofectamine 2000 and Fugene HD did not allow transfection of DNA (Fig. 2*G*). In fact, only PEI 25k and PEI 40k could mediate transfection, the former reaching transfection efficiency of ∼2% (at 8:1 ratio to DNA) and the latter ∼0.5% efficiency (at 9:1 ratio to DNA). For mRNA transfection, Lipofectamine 2000 showed ∼5% transfection efficiency when applied at 2:1 ratio to mRNA. Fugene HD could not mediate mRNA transfection. While PEI 40k could not mediate mRNA transfection, PEI 25k allowed transfection efficiency of ∼2% when used at 3:1 ratio to mRNA. Of the DOPE:DOTAP mixes, 1:1 yielded the highest transfection efficiency (∼4.5%) when mixed at 15:1 ratio to mRNA. The 1:1 ratio produced the highest transfection efficiency for DOPE:DOTMA, reaching ∼6% at 3:1 reagent:mRNA ratio.

In HUH-7 cells, Lipofectamine 2000 showed efficiency to increase with the reagent-to-DNA ratio, reaching ∼30% transfection efficiency at 6:1 ratio (Fig. 2*H*). Fugene HD showed a similar trend but reached only ∼6% maximal transfection efficiency at the reagent:DNA ratios tested. PEI 25k showed transfection efficiencies ∼30% over a broad range of reagent:DNA ratios, 5:1 ratio demonstrating the highest efficiency (∼32%). PEI 40k showed similar the best performance, reaching over 50% transfection efficiency with reagent ratios 7:1 to 9:1. DOPE:DOTAP, irrespective of the ratio mixed, showed poor DNA transfection efficiency, reaching <5% maximal transfection rate. DOPE:DOTMA mixes performed better, with 2:1 DOPE:DOTMA mix showing the highest transfection efficiency (∼25%) at 9:1 reagent:DNA ratio. For mRNA transfection, Lipofectamine 2000 reached >80% transfection efficacy at 2:1 and 4:1 reagent:mRNA ratios and almost 70% at 6:1 ratio. Fugene HD, PEI 25k, and PEI 40k mediated mRNA transfection with respective efficiencies of ∼0%, ∼2.5%, and ∼3.5%. DOPE:DOTAP mixed at 0.5:1 ratio provided the highest mRNA transfection efficiency, with >50% transfection rate reached at 15:1 reagent:mRNA ratio. For DOPE:DOTMA, the 1:1 and 2:1 mixes performed the best with >90% transfection efficiency reached using 1:1 DOPE:DOTMA mixed with mRNA at 6:1 or 9:1 ratio.

In BHK-21 cells, we achieved approximately 30% transfection efficacy with Lipofectamine 2000 at all reagent-to-pDNA ratios tested. With the other positive control, Fugene HD, the transfection efficiency increased toward higher ratios, reaching approximately 30% at the highest reagent to pDNA ratio (Fig. 2*I*). PEI 25k demonstrated the 40% transfection efficiency when used at 3:1 ratio to pDNA, the other ratios demonstrated lower efficacy. PEI 40k showed good transfection efficiency over a broad range of reagent to pDNA ratios (5:1–8:1), allowing transfection of 55% of the cells at 8:1 ratio. For DOTAP reagents, the reagent mixed with DOPE at 1:1 ratio demonstrated the highest transfection rates with reagent to pDNA ratios between 6:1 and 15:1, topping at approximately 30% transfection efficacy. DOTMA reagents with DOPE:DOTMA ratios 1:1 and 2:1 reached approximately 30% transfection efficacy, with 2:1 ratio demonstrating a broader range of reagent to pDNA ratios (9:1–15:1) ratio yielding high efficiency. In the case of mRNA, Lipofectamine 2000 demonstrated high transfection efficiency over all tested reagent to mRNA ratios, with a maximum of approximately 75% of cells being transfected at 4:1 ratio (Fig. 2*A*). Fugene HD did not allow mRNA transfection at the tested concentrations. The polyamines PEI 25k and PEI 40k showed negligible mRNA transfection efficacy, reaching maximum of approximately 2% of transfected cells. Of the DOPE:DOTAP mixes, 0.5:1 ratio showed an increasing efficiency at higher reagent concentration, reaching the highest transfection rate of approximately 40% at reagent-to-mRNA ratios of 15:1. DOPE:DOTAP mixed at 1:1 ratio showed transfection rate of >30% over a broad range of reagent-to-mRNA ratios (3:1–9:1), whereas DOPE:DOTAP mixed at 2:1 showed the lowest mRNA transfection rate at the concentration range tested. With DOTMA, the 1:1 and 2:1 DOPE:DOTMA mixes showed the highest mRNA transfection efficiencies, with the highest transfection rate of 55% observed with DOPE:DOTMA 1:1 mixed at 3:1 ratio with the mRNA.

In BVK-168 cells, Lipofectamine 2000 showed ∼2% transfection efficiency when applied at 6:1 ratio to DNA, and the other commercial reagent, Fugene HD, reached the highest efficiency (∼0.5%) at 3:1 ratio to DNA (Fig. 2*J*). Both PEI 25k and PEI 40k, showed highest transfection efficiency at 9:1 reagent:DNA, respectively reaching ∼2% and ∼3% efficiencies. Of the DOPE:DOTAP mixes, 0.5:1 showed the highest transfection efficiency (∼0.5%) at 15:1 ratio to DNA. Of the DOPE:DOTMA mixes, 1:1 yielded the highest transfection efficiency, reaching ∼2% at 15:1 ratio to DNA. For mRNA transfection, Lipofectamine 2000 showed ∼10% transfection efficiency at all concentrations tested. Fugene HD could not mediate mRNA transfection. PEI 25k showed ∼2% transfection efficiency when mixed 5:1 with mRNA, while PEI 40k demonstrated ∼5% efficiency when applied at 5:1 ratio to mRNA. Of the DOPE:DOTAP mixes, both 0.5:1 and 1:1 showed ∼45 to 50% transfection efficiencies, 0.5:1 reaching ∼45% when applied at 6:1 ratio to mRNA and 1:1 reaching ∼50% efficiency at 15:1 ratio to mRNA. Of the DOPE:DOTMA mixes, 2:1 produced the best results, reaching ∼30% transfection efficiency at 6:1 and ∼25% at 9:1 to 15:1 reagent: mRNA ratios.

In CHO cells, Lipofectamine 2000 showed transfection efficacy to rise with increasing reagent-to-DNA ratio, with the highest efficacy of ∼20% recorded at 6:1 tested (Fig. 2*K*). Fugene HD demonstrated a similar trend with a maximum transfection efficacy of ∼35%. PEI 25k showed variation in the efficacy over the range of reagent-to-DNA ratios tested, with the highest efficiency (∼20%) at a 3:1 ratio, whereas PEI 40k demonstrated a trend of efficacy increasing with reagent-to-DNA ratio until reaching the maximal efficacy (∼25%) at an 8:1 ratio. DOPE:DOTAP and DOPE:DOTMA both reached ∼7.5% DNA transfection efficacy, for DOPE:DOTAP 1:1 mix and for DOPE:DOTMA 0.5:1 mix produced the best result (both at 9:1 reagent:DNA). Lipofectamine 2000 performed the best also in mRNA transfection of CHO cells, reaching ∼65% transfection efficiency at a 4:1 reagent-to-mRNA ratio. Fugene HD, PEI 25k, and PEI 40k mediated mRNA transfection with respective efficiencies of ∼0%, ∼1.5%, and ∼2.5%. DOPE:DOTAP worked best at 0.5:1 and 1:1 ratios, reaching the highest mRNA transfection efficacy of ∼12% at 12:1 (or 15:1 for 0.5:1 DOPE:DOTAP) reagent:mRNA ratio. DOPE:DOTMA demonstrated a similar behavior with the highest transfection efficacy (∼14% 15:1 reagent:mRNA) at 1:1 ratio, but with slightly lower mRNA efficacy at 0.5:1 ratio than DOPE:DOTAP (Fig. 2*K*).

In I/1Ki cells, Lipofectamine 2000 showed DNA increasing transfection efficiency with higher reagent concentrations, reaching ∼90% efficiency when used at 6:1 reagent:DNA ratio (Fig. 2*L*). Fugene HD demonstrated similar dependency to the reagent concentration, and the highest ratio tested, 4.5:1 reagent:DNA, showed >40% transfection efficiency. Both PEI 25k and PEI 40k yielded transfection efficiencies <5% even at the highest reagent: DNA ratios. Of the DOPE: DOTAP mixes, only 1:1 mix showed some transfection efficiency (<5%). Similarly, the 1:1 mix performed the best for DOPE:DOTMA, yielded transfection efficiency (∼10%) at 9:1 reagent:DNA ratio. For mRNA transfection, Lipofectamine 2000 demonstrated transfection efficiency of >80% at 4:1 reagent:mRNA ratio, and >70% even at the highest concentration tested. Fugene HD could not mediate transfection at the concentration range tested. PEI 25k showed transfection efficiency of ∼25% when applied at 4:1 reagent:mRNA ratio, and the efficiency declined with increasing reagent concentration. With PEI 40k, 2:1 reagent:mRNA ratio yielded highest transfection efficiency >5%, and the higher reagent concentration showed a decreasing efficiency. Of the DOPE:DOTAP mixes, 1:1 showed the best performance, yielding >5% transfection efficiency at 3:1 reagent:mRNA ratio. With DOPE:DOTMA, the 0.5:1 mix performed the best, yielding <5% transfection efficiency when mixed 1:1 with the mRNA.

In Vero E6 cells, Lipofectamine 2000 showed increasing DNA transfection efficiency with higher reagent concentrations, reaching ∼15% efficiency at 6:1 reagent:DNA ratio (Fig. 2*M*). Fugene HD performed best at 3:1 reagent:DNA ratio, yielding >5% transfection efficiency. PEI 25k showed the highest transfection efficiency (<5%) at 3:1 to 4:1 reagent:DNA ratio, while ratios of 7:1 to 9:1 produced the highest efficiencies (>5%) for PEI 40k mediated transfection. DOPE:DOTAP 0.5:1 mix demonstrated ≥10% transfection efficiency at reagent:DNA ratios of 12:1 and 15, and the DOPE:DOTAP 1:1 mix showed similar efficiency at ratios 6:1, 9:1, and 15:1. For DOPE: DOTMA, the 1:1 mix produced the highest transfection efficiency (<10%) at reagent: DNA ratio of 12:1, whereas 2:1 DOPE:DOTMA showed similar efficiency for reagent:DNA ratios between 6:1 and 12:1. For mRNA transfection, Lipofectamine 2000 showed transfection efficiency of ∼5% for the concentrations testes, and the other commercial reagent, Fugene HD, did not mediate mRNA transfection. While PEI 25k demonstrated transfection efficiency of <5%, PEI 40k reached >10% transfection efficiency at reagent:mRNA ratios between 6:1 and 8:1. The DOPE:DOTAP mixes showed similar transfection efficiencies, 0.5:1 and 1:1 DOPE:DOTAP mixes reached almost 50% transfection rate at 6:1 reagent:mRNA ratio, whereas 9:1 ratio of DOPE:DOTAP 2:1 mix produced similar efficiency. The DOPE:DOTMA mixes 1:1 and 2:1 showed the highest transfection efficiencies, with 1:1 mix reagent:mRNA ratios 3:1 and 6:1 yielded almost 80% transfection efficiency, and with 2:1 mix 6:1 ratio showed ∼70% transfection efficiency.

In XTC cells, Lipofectamine 2000 yielded a transfection efficiency of ∼1 to 2% (Fig. 2*N*). Fugene HD reached ∼5% transfection efficiency when used at 3:1 and 4.5:1 reagent:DNA ratio. The other reagents tested showed transfection efficiencies of only ∼1% or less. For mRNA transfection, Lipofectamine 2000 demonstrated ∼35% efficiency at 2:1 reagent:mRNA ratio, and >30% efficiency at 4:1 and 6:1 ratio. Fugene HD showed mRNA transfection efficiency of ∼10% at the highest reagent:mRNA ratio (4.5:1). PEI 25k showed transfection efficiency of ∼25% with reagent:mRNA ratios between 4:1 to 9:1. PEI 40k showed the highest mRNA transfection efficiencies, reaching 45% to ∼50% efficiency with 7:1 to 9:1 reagent:mRNA ratios, and was the most effective among all tested reagents, achieving transfection efficiencies between at higher reagent-to-mRNA ratios. Of the DOPE:DOTAP mixes, 1:1 mix showed the best performance, reaching ∼5% transfection efficiency at 1:1 and 3:1 ratio to mRNA. Of the DOPE:DOTMA mixes, 2:1 showed the highest performance, reaching ∼20% transfection efficiency at 3:1 and 6:1 reagent:mRNA ratio.

### Cytotoxicity of the transfection reagents

**Figure 3:**
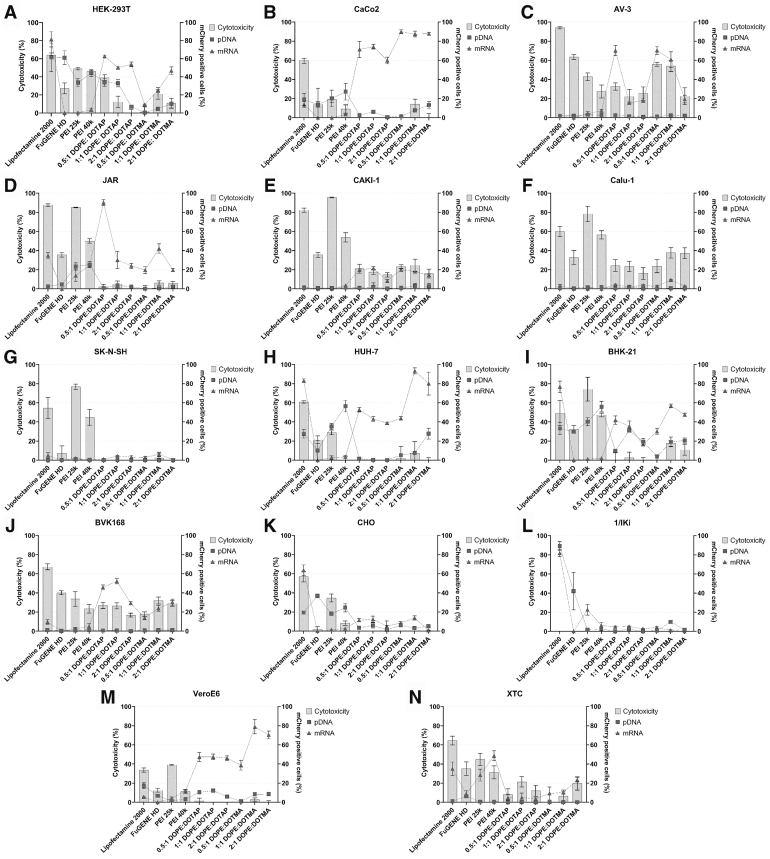
**Transfection reagent cytotoxicity in relation to transfection efficiency.** The cytotoxicity of the six transfection reagents was evaluated across 14 cell lines: (*A*) HEK-293T, (*B*) Caco-2, (C) AV3, (*D*) JAR, (*E*) Caki-1, (*F*) Calu-1, (*G*) SK-N-SH, (*H*) Huh-7, (*I*) BHK-21, (*J*) BVK-168, (*K*) CHO, (*L*) I/1Ki, (*M*) Vero E6, and (*N*) XTC. Cell viability was determined based on the percentage of cytotoxicity and correlated with transfection efficiency for both pDNA and mRNA using the respective reagents. Results are presented as mean ± SD from four technical replicates. pDNA, plasmid DNA.

Following the evaluation of transfection efficiencies, we assessed the cytotoxicity of each reagent at the highest concentration used during the transfection experiments across 14 cell lines: HEK-293T, Caco-2, AV3, JAR, Caki-1, Calu-1, SK-N-SH, Huh-7, BHK-21, BVK-168, CHO, I/1Ki, Vero E6, and XTC. Instead of testing the full concentration range, we chose to test the effect of the reagents at the highest concentration applied in the former experiment. We used pDNA for the transfection and CellTiter-Glo 2.0 cell viability assay for evaluating the cytotoxicity of the reagents 48 h posttransfection through comparison to cells grown under the same conditions without DNA and transfection reagent addition. Along the cytotoxicity, we plotted for each cell line the highest transfection efficiency (as shown in Fig. 2) achieved for both pDNA and mRNA with the respective reagent (Fig. 3).

In HEK-293T cells, Lipofectamine 2000 induced the highest cytotoxicity (>60%), followed by PEI 25k and PEI 40k (>40%). DOPE:DOTAP (0.5:1) exhibited ∼40% cytotoxicity, and Fugene HD ∼30%. DOPE:DOTMA and DOPE:DOTAP at higher DOPE ratios showed the lowest toxicity. Comparison of cytotoxicity *versus* transfection efficiency suggests DOPE:DOTAP as the ideal transfection reagent for mRNA, and Fugene HD for pDNA (Fig. 3*A*).

In Caco-2 cells, Lipofectamine 2000 and PEI 25K caused the highest cytotoxicity (60% and 20%, respectively). Based on cytotoxicity *versus* transfection efficiency, the DOPE:DOTMA mixes appear ideal for mRNA delivery while PEI 40K appears most suitable for pDNA (Fig. 3*B*).

In AV3 cells, Lipofectamine 2000 (>90%), Fugene HD, and DOTMA at lower DOPE ratios (∼60%) showed highest cytotoxicity. While the experiments showed negligible pDNA transfection efficacy, PEI 25k and PEI 40k allowed some pDNA transfection. In relation to cytotoxicity DOPE:DOTAP at 0.5:1 provided the best mRNA transfection performance (Fig. 3*C*).

In JAR cells, Lipofectamine 2000 and PEI 25k showed high cytotoxicity (∼90%), and PEI 40k (50%) and Fugene HD (>35%) showed moderate cytotoxicity, while the other reagents demonstrated rather low cytotoxicities (<10%). Based on efficiency and cytotoxicity PEI 40k appeared most suitable for pDNA transfection and DOPE:DOTAP at 0.5:1 for mRNA transfection (Fig. 3*D*).

In Caki-1 cells, PEI 25k (>90%) and Lipofectamine 2000 (∼80%) showed highest cytotoxicity, followed by PEI 40k (∼50%) and Fugene HD (∼35%). While pDNA transfection was negligible across the reagents, 1:1 DOPE:DOTMA mix allowed some transfection at relatively low cytotoxicity. DOPE:DOTAP mixed at 1:1 showed the best mRNA transfection efficacy in relation to cytotoxicity (Fig. 3*E*).

In Calu-1 cells, PEI 25k showed highest cytotoxicity (∼80%), followed by Lipofectamine 2000 and PEI 40k (both ∼60%). Only DOPE:DOTMA (1:1) showed some mRNA transfection efficiency (∼10%), but none of the reagents mediated pDNA transfection (Fig. 3*F*).

In SK-N-SH cells, PEI 25k, Lipofectamine 2000, and PEI 40K caused significant cytotoxicity (80%, 55%, and 40%, respectively). None of the reagents could mediate pDNA transfection, however, DOPE:DOTMA (1:1) could drive mRNA transfection (∼10% efficiency) with little cytopathic effect (Fig. 3*G*).

In Huh-7 cells, Lipofectamine 2000, PEI 25k, and Fugene HD showed the highest cytotoxic effect (∼60%, ∼30%, and ∼20%). PEI 40k demonstrated good pDNA transfection efficiency (∼60%) with practically no cytopathic effect. For mRNA transfection, DOPE:DOTMA at 1:1 ratio showed >90% transfection efficiency with little (∼10%) cytopathic effect (Fig. 3*H*).

In BHK-21 cells, PEI 25k, Lipofectamine 2000, PEI 40k, and Fugene HD showed high cytotoxicity (∼75%, ∼50%, ∼50%, and ∼35%, respectively). While these reagents provided the highest pDNA transfection efficacies, DOPE:DOTAP at 1:1 ratio appeared to provide good transfection (∼35%) efficacy almost without cytotoxic effects. DOPE:DOTAP at 0.5:1 mediated mRNA transfection quite efficiently (∼40%) without cytopathic effects, suggesting it to more suitable than Lipofectamine 2000 and DOPE:DOTMA at 1:1 (mRNA transfection efficacies ∼75% and ∼60%) if low cytotoxicity is required (Fig. 3*I*).

In BVK-168 cells, Lipofectamine 2000 showed cytotoxicity of almost 70%; however, all other reagents demonstrated also significant cytotoxicity (between ∼20% and ∼40%). None of the reagents mediated pDNA transfection. DOPE:DOTAP 1:1 mix showed the highest mRNA transfection efficiency (∼55%) (Fig. 3*J*).

In CHO cells, Lipofectamine 2000, PEI 25k, and PEI 40k showed respective cytotoxicity values of ∼60%, ∼35%, and ∼10%, while the other reagents appeared noncytotoxic. Fugene HD performed the best for pDNA transfection (∼40% efficiency). While Lipofectamine 2000 showed great mRNA transfection efficacy (>60%), DOPE:DOTAP and DOPE:DOTMA mixes reaching ∼15% mRNA transfection efficiency might be an option if low cytotoxicity is required (Fig. 3*K*).

In I/1Ki cells, none of the tested reagents showed cytotoxicity. Lipofectamine 2000 demonstrated high efficiency for both pDNA and mRNA transfection (>80%), whereas the other reagents did not appear to provide reproducible transfection results for pDNA or mRNA (Fig. 3*L*).

In Vero E6 cells, Lipofectamine 2000, PEI 25k, Fugene HD, and PEI 40k showed respective cytotoxicity values of ∼35%, ∼40%, ∼10%, and ∼10%, while the other reagents appeared noncytotoxic. While Lipofectamine 2000 performed best for pDNA transfection (∼20% efficiency), DOPE:DOTAP at 1:1 ratio appears as a better choice with ∼15% pDNA transfection efficiency due to lower cytotoxicity. DOPE:DOTMA at 1:1 ratio showed the highest mRNA transfection efficiency (∼80%) (Fig. 3*M*).

In XTC cells, Lipofectamine 2000 showed the highest cytotoxicity (∼65%), followed by PEI 25k, Fugene HD, and PEI 40k with respective cytotoxicities of ∼45%, ∼35%, and ∼30%. Only Fugene HD showed some pDNA transfection efficiency (∼10%), and PEI 40k showed the best mRNA transfection efficiency (∼50%) (Fig. 3*N*).

### Stability of nucleic acid–transfection reagent complexes

**Figure 4:**
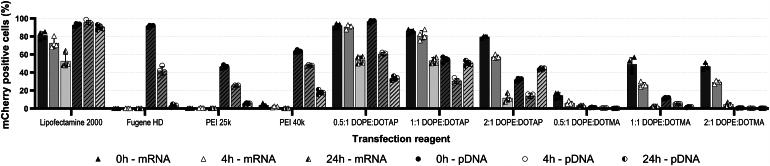
**Stability of nucleic acid–reagent complexes during storage at 4 °C.** Stability analysis of nucleic acid–reagent complexes formed with six transfection reagents—Lipofectamine 2000, Fugene HD, PEI 25k, PEI 40k, DOPE:DOTAP, and DOPE:DOTMA—evaluated in HEK-293T cells at 0, 4, and 24 h of incubation of transfection complexes with pDNA and mRNA. Transfection efficiency was assessed at 48 h posttransfection to determine the retention of activity. Bar graphs display the mean ± SD from four independent technical replicates, with superimposed scatter plots illustrating the reproducibility of individual replicate measurements. DOPE, dioleoylphosphatidylethanolamine; DOTAP, 1, 2-dioleoyl-3-trimethylammonium-propane; DOTMA, N-[1-(2,3-dioleyloxy)propyl]-N,N,N-trimethylammonium chloride; pDNA, plasmid DNA.

Finally, we studied the stability of the transfection reagent:nucleic acid complexes through transfecting HEK-293T cells with pDNA or mRNA complexed for 0, 4, or 24 h (stored at 4 °C) earlier with each reagent at the ratio yielding the highest transfection efficiency (Fig. 2). For pDNA, Lipofectamine 2000 yielded ∼100% transfection efficiency up to 24 h (Fig. 4). With Fugene HD, we detected ∼100% efficiency at complexes prepared 4 h before transfection; however, the complexes stored for 24 h showed ∼90% reduction in transfection efficiency. While PEI 25k showed the transfection efficiency to decrease dramatically from 85% to ∼60% in 4 h, and to ∼25% in 24 h, the complexes of PEI 40k appeared more stable and provided respective efficiencies of ∼65%, >80%, and >80%. DOPE:DOTAP mix 0.5:1 showed the transfection efficiency to decrease from ∼95% to ∼60% in 4 h and down to ∼35% in 24 h. Curiously, both 1:1 and 2:1 DOPE:DOTAP combinations showed the efficiency to decrease during the first 4 h of incubation and to recover during prolonged incubation. DOPE:DOTMA mixes showed the ratio not to affect the complex stability, the efficiency decreasing during the storage, for example, DOPE:DOTMA 0.5:1 mix showed ∼30%, ∼5%, and <5% transfection efficiencies for complexes stored 0, 4, and 24 h.

For mRNA, Lipofectamine 2000 showed transfection efficiencies of ∼90%, ∼84%, and ∼76% for complexes stored at 0, 4, or 24 h, respectively. In alignment with the initial testing (Fig. 2), Fugene HD and PEI 25K could not mediate mRNA transfection to a meaningful extent. PEI 40k:mRNA complexes showed transfection efficiency to fall from ∼4.8% to ∼1% in 4 h and to ∼0.7% in 24 h. Both DOPE:DOTAP and DOPE:DOTMA mixes showed transfection efficiency to decrease during storage of the complexes for up to 24 h. While the efficiency decrease was rather steady for most combinations, the DOPE:DOTAP mixes 0.5:1 and 1:1, and DOPE: DOTMA 1:1 and 2:1 showed the complexes to remain stable for 4 h when stored at 4 °C.

## Discussion

Transfection is a pivotal molecular biology technique, essential in applications ranging from protein expression and gene editing or silencing to drug discovery and vaccine development. However, the transfection efficiency varies considerably between cell lines and the reagents used in the process, making optimization and reagent selection crucial for success. The commercially available reagent formulations are highly effective in mediating transfection, and they are often the method of choice, even though they may come with a relatively high price, thereby posing challenges in upscaling the experiments. In addition to the ready formulations, researchers often employ polyamines or polycations, respectively exemplified by, for example, PEI and DOTAP, to prepare “in-house” reagents for their specific needs and use cases. The transfection efficiencies of the reagents have usually been studied in some commonly used cell lines, and studies evaluating the transfection efficiencies of both DNA and RNA are rare. This motivated us to perform a systematic study on the pDNA and mRNA transfection efficiency of commonly used “in-house” reagents PEI 25k, PEI 40k, DOPE:DOTAP, and DOPE:DOTMA across 14 cell lines. We selected two commercially available formulations, Lipofectamine 2000 and Fugene HD, to allow comparison to a positive control but not to perform optimization-based evaluations against these reagents.

The commercial reagents, Lipofectamine 2000 and Fugene HD, performed well in a broad range of cell lines for pDNA transfection, the primary use case of these reagents. In addition, Lipofectamine 2000 efficiently mediated mRNA transfection on multiple cell lines. In contrast, as expected based on the product manual, Fugene HD could not mediate mRNA transfection in most cell lines. Curiously, the reagent did allow mRNA transfection in an amphibian cell line, suggesting some potential applicability in RNA delivery. Among the tested reagents, DOPE:DOTAP and DOPE:DOTMA formulations emerged as highly effective, particularly for challenging cell lines such as AV3, Caco2, and Vero-E6, where conventional reagents like Lipofectamine 2000 demonstrated comparatively lower performance.

The transfection efficiency observed with DOPE:DOTAP and DOPE:DOTMA formulations for pDNA and mRNA delivery, particularly in challenging cell lines, is likely primarily attributed to the interplay between lipid molecular architecture, membrane dynamics, and the physicochemical synergy between cationic and helper lipids. DOTAP and DOTMA, as permanently charged cationic lipids under physiological pH, mediate strong electrostatic interactions with anionic nucleic acids, enabling efficient complex formation and cellular uptake. However, their transfection capabilities and complex stability are markedly potentiated by the inclusion of the fusogenic helper lipid DOPE. Owing to its intrinsic cone-shaped geometry derived from unsaturated oleoyl chains, DOPE promotes the formation of the nonlamellar inverted hexagonal (HII) phase under endosomal acidic pH conditions. This phase transition disrupts endosomal membranes, thereby enhancing endosomal escape and facilitating the cytosolic release of nucleic acids. Thus, the combination of membrane-condensing cationic lipids with the membrane-destabilizing properties of DOPE yields lipoplexes with optimized intracellular trafficking and delivery performance (46, 47, 48).

The comprehensive assessment of transfection reagent cytotoxicity across 14 distinct cell lines revealed significant variability, highlighting the necessity of reagent selection tailored to cell type-specific responses. Among commercial reagents, Lipofectamine 2000 (a 1:3 mix of DOPE and 2,3-dioleyloxy-N-[2(sperminecarboxamido)ethyl]-N,N-dimethyl-1-propanaminium (DOSPA) exhibited the highest cytotoxicity (30%–95%), whereas Fugene HD demonstrated comparatively lower toxicity (15%–65%). Similarly, polyamines PEI 25k and PEI 40k showed broad cytotoxicity ranges (∼20%–95% and ∼0%–55%, respectively), paralleling the impact of commercial reagents on cell viability. In contrast, DOPE:DOTAP and DOPE:DOTMA exhibited lower cytotoxicity, especially at higher DOPE ratios. DOPE-containing lipid systems are associated with significantly lower cellular toxicity than conventional transfection reagents (49), offering a critical advantage when working with sensitive or otherwise refractory cell types. DOPE:DOTAP formulations (0.5:1, 1:1, and 2:1) displayed minimal cytotoxic effects (∼0%–40%, ∼0%–25%, and ∼0%–25%, respectively), while DOPE:DOTMA formulations exhibited slightly higher cytotoxicity (up to ∼55%). DOTAP, which contains degradable ester linkages between its cationic head group and hydrophobic lipid chains, exhibits slightly enhanced transfection efficiency and lower cytotoxicity than DOTMA when coformulated with DOPE. The presence of ester bonds facilitates controlled degradation, reducing prolonged cellular stress while maintaining efficient nucleic acid delivery (46). Notably, AV3, Caki-1, Calu-1, and BVK-168 displayed high sensitivity to all tested reagents, suggesting an inherent susceptibility to transfection-induced cellular stress. In contrast, the remaining cell lines tolerated DOPE-based formulations better than polyamines and commercial reagents. The high charge density of Lipofectamine 2000 and PEI leads to strong electrostatic interactions with cell membranes, resulting in oxidative stress, mitochondrial dysfunction, and caspase-mediated apoptosis (50, 51). In contrast, DOTAP and DOTMA, when combined with DOPE, achieve a balanced charge ratio, minimizing excessive electrostatic interactions that could compromise cell viability (52).

The stability of transfection reagent:nucleic acid complexes is crucial for maintaining high transfection efficiencies over time. Our results show distinct stability profiles for pDNA and mRNA complexes governed by their structural and physicochemical properties. For pDNA transfection, Lipofectamine 2000 exhibited a remarkable stability, maintaining near-complete efficiency even after 24 h, likely due to its well-optimized lipid composition and polycationic nature of the cationic lipid DOSPA that helps to preserve electrostatic interactions. In contrast, Fugene HD and PEI 25k showed significant degradation in transfection efficiency. Fugene HD complexes experienced a sharp decline after 4 h and PEI 25k demonstrated instability at all time points. The higher molecular weight PEI 40k, however, conferred greater stability, potentially due to stronger electrostatic interactions that sustain complex integrity (53). Similarly, DOPE:DOTAP and DOPE:DOTMA formulations exhibited progressive declines in efficiency, with variations in lipid ratios affecting initial transfection performance and long-term stability. In the case of mRNA, the slightly lower stability observed across all formulations reflects the inherent susceptibility of ssRNA to hydrolytic degradation and complex dissociation. Lipofectamine 2000 remained the most effective reagent, but its efficiency declined over time, indicating moderate instability. PEI 40k complexes lost efficacy rapidly, whereas DOPE:DOTAP formulations exhibited relatively stable transfection efficiencies during the first 4 h at 4 °C, suggesting a potential strategy for short-term storage.

These results highlight the necessity of tailoring transfection reagents to the specific nucleic acid type. The superior stability of pDNA complexes can be attributed to their double-stranded nature, which enables more robust electrostatic interactions with cationic lipids and polyamines, ensuring prolonged complex integrity. The increased stability of Lipofectamine, which consists of a DOPE:DOSPA lipid formulation, compared to DOPE:DOTAP and DOPE:DOTMA systems, results from fundamental differences in the chemical structure and biophysical properties of the constituent cationic lipids. DOSPA has a spermine-based polyamine head group that facilitates multivalent electrostatic interactions with nucleic acids, providing a clear advantage over the monovalent quaternary ammonium groups of DOTAP and DOTMA. This multivalency encourages more effective nucleic acid condensation and produces lipoplexes with improved resistance to dissociation under physiological conditions. Furthermore, DOSPA, along with the fusogenic helper lipid DOPE, promotes the formation of highly ordered and compact lipid bilayers, which enhances the structural integrity and serum stability of the complexes. In contrast, DOTAP- and DOTMA-based formulations generally yield fewer stable assemblies that are susceptible to premature disintegration and decreased transfection performance in biologically relevant environments (28, 31, 54). Moreover, the greater stability of PEI 40K than PEI 25K is primarily due to its higher molecular weight, which enhances nucleic acid binding affinity and complex condensation through increased charge density and entanglement. However, despite these advantages, PEI formulations are generally less stable than cationic lipid formulations incorporating helper lipids such as DOPE. This reduced stability arises from the inherent physicochemical properties of PEI, which form less compact and more heterogeneous polyplexes that are prone to aggregation and premature disassembly under physiological conditions (15, 53).

In conclusion, our study underscores the potential of cationic lipid, DOTAP, and DOTMA-based formulations, as promising lower cost alternatives for both pDNA and mRNA delivery into cultured cells with only moderate cytotoxic effect in most cell lines. On the other hand, the PEIs tested showed to be suitable for pDNA delivery into some hard-to-transfect cell lines, but with the cost of cytotoxic effects, highlighting the need for tailored protocols and transfection formulations for refractory cell lines. The stability of DOPE:DOTAP and DOPE:DOTMA complexes also supports their utility in applications requiring short-term storage, particularly for mRNA delivery, where commercial reagents like Lipofectamine 2000 exhibit progressive efficiency loss. These findings indicate that high transfection efficiency can be achieved without reliance on expensive reagents, as well-optimized lipid-based alternatives provide a strategic balance between cost-effectiveness and performance. The adaptability of DOPE-lipid formulations to specific cell types and nucleic acid delivery requirements enhances experimental flexibility, facilitating wider adoption in molecular and cellular research. Overall, our study provides a broad go-to reference for selecting lower cost transfection reagents based on cell line and nucleic acid type to allow faster protocol optimization and advance the accessibility of transfection technologies.

## Experimental procedures

### Cell lines and culturing conditions

Cell lines originating from human, monkey, frog, snake, and rodent tissues, as detailed in Table 1, were cultured in T-75 cell culture flasks (Greiner, lot: E24103E3) until use. The cell culture media were minimum essential medium eagle (MEM, Sigma-Aldrich, M2279-500 Ml), Dulbecco's modified Eagle's medium (DMEM high gl., Sigma-Aldrich, D6546-500 ML), Dulbecco's modified Eagle's medium (low gl., Sigma-Aldrich, D5546-500 Ml), RPMI medium 1640 1X (Gibco, 31870-025), and Leibovitz's (1X) L-15 medium (Gibco, 11415-064). The supplements were fetal bovine serum (FBS, Gibco, A526701), 200 mM L-glutamine (Sigma-Aldrich, G7513-100 Ml), penicillin-streptomycin Solution 100X (Biowest, L0022-100) and nonessential amino acids 100X (Gibco, 11140-050), as detailed in Table 1. For HEK-293T, XTC, and I/1Ki cells, the culture plates were coated by incubating the plates overnight at 4 °C with 50 μl/well of 0.1 mg/ml rat tail collagen type I (Corning, Ref 354236) diluted in 25 mM acetic acid. The cell lines have been tested negative for *mycoplasma* periodically.

**Table 1:** Cell lines used in the study

Cell line	Origin	Culture medium	Cells/well	Incubation
Human embryonic kidney 293T (HEK-293T)	Human	DMEM high glucose; 10% FBS, 2 mM L-glutamine, 100 U/ml penicillin, 100 μg/ml streptomycin	15,000	37 °C
Colon adenocarcinoma-2 (Caco-2)	Human	MEM, 20% FBS, 1xNEAA, 100 U/ml penicillin, 100 μg/ml streptomycin	22,000	37 °C
Human cervix carcinoma (AV3)	Human	MEM, 10% FBS, 2 mM L-glutamine, 100 U/ml penicillin, 100 μg/ml streptomycin	15,000	37 °C
Human choriocarcinoma (JAR)	Human	RPMI; 10% FBS, 2 mM L-glutamine, 100 U/ml penicillin, 100 μg/ml streptomycin	30,000	37 °C
Clear cell carcinoma kidney-1 (Caki-1)	Human	RPMI; 10% FBS, 2 mM L-glutamine, 100 U/ml penicillin, 100 μg/ml streptomycin	20,000	37 °C
Human lung carcinoma (Calu-1)	Human	RPMI; 10% FBS, 2 mM L-glutamine, 100 U/ml penicillin, 100 μg/ml streptomycin	25,000	37 °C
Human neuroblastoma (SK-N-SH)	Human	DMEM low glucose; 10% FBS, 2 mM L-glutamine, 100 U/ml penicillin, 100 μg/ml streptomycin	30,000	37 °C
Human hepato cellular carcinoma (Huh-7)	Human	DMEM low glucose; 10% FBS, 2 mM L-glutamine, 100 U/ml penicillin, 100 μg/ml streptomycin	20,000	37 °C
Baby hamster kidney 21 (BHK-21)	Rodent	MEM, 10% FBS, 2 mM L-glutamine, 100 U/ml penicillin, 100 μg/ml streptomycin	15,000	37 °C
Bank vole kidney 168 (BVK-168)	Rodent	DMEM high glucose, 10% FBS, 2 mM L-glutamine, 100 U/ml penicillin, 100 μg/ml streptomycin	30,000	37 °C
Chinese hamster ovary (CHO)	Rodent	DMEM high glucose; 10% FBS, 2 mM L-glutamine, 100 U/ml penicillin, 100 μg/ml streptomycin	15,000	37 °C
*Boa constrictor* kidney (I/1Ki)	Snake	MEM, 10% FBS, 2 mM L-glutamine, 100 U/ml penicillin, 100 μg/ml streptomycin	30,000	28 °C
African green monkey kidney (Vero E6)	Monkey	MEM, 10% FBS, 2 mM L-glutamine, 100 U/ml penicillin, 100 μg/ml streptomycin	15,000	37 °C
*Xenopus* tadpole cells (XTC)	Frog	70% Leibovitz L-15, 20% dH_2_O, 10% FBS, 2 mM L-glutamine, 100 U/ml penicillin, 100 μg/ml Streptomycin	30,000	28 °C

All cell lines were obtained from ATCC except BVK-168 (55) and I/1Ki (56).

DMEM, Dulbecco's modified Eagle's medium; FBS, fetal bovine serum; NEAA, nonessential amino acid.

### Plasmid propagation and mRNA *in vitro* transcription

The plasmid carrying mCherry was constructed by subcloning a synthetic gene (GeneArt) flanked by EcoRI and XhoI restriction sites into pCAGGS vector at the respective sites. FastDigest restriction enzymes and T4 ligase used according to the manufacturer’s protocol (Thermo Fisher Scientific) served for the cloning. After selecting a clone carrying the insert, as confirmed by Sanger sequencing, the plasmid amplification was achieved using ZymoPURE II plasmid maxiprep kit (Zymo Research). The pDNA was utilized as a template for PCR to amplify the target gene containing the T7 promoter, with forward primer: 5′-TAATACGACTCACTATAGGCCACCATGGTGTCCAAGGGCGAG-3′ and reverse primer: 5′-TTAGGTGGAGTGCCTGCCCTCG-3′. The PCR protocol with Q5 High-Fidelity 2x master mix (New England BioLabs) comprised initial denaturation at 98 °C for 2 min, two cycles of denaturation at 98 °C for 1 s, annealing at 55 °C for 1 s, and extension at 72 °C for 10 s, followed by 34 cycles of denaturation at 98 °C for 1 s, annealing at 68 °C for 1 s, extension at 72 °C for 10 s, followed by a final extension at 72 °C for 2 min. The PCR products were purified using the NucleoSpin Gel and PCR Clean-up kit (Macherey-Nagel, 740609) following the manufacturer's protocol. Subsequently, *in vitro* transcription of mCherry mRNA was conducted using HiScribe T7 ARCA mRNA Kit (New England BioLabs, E2060S) following the manufacturer's instructions with the PCR product as a template and subsequent polyA tailing. The purified DNA, pDNA, and mRNA were quantified using a NanoDrop 2000 spectrophotometer (Thermo Fisher Scientific).

### Preparation of transfection reagents

PEI 25k and PEI 40k were obtained from Polysciences. We prepared 1 mg/ml stocks of the respective reagent in MilliQ water, used heated (50 °C) water bath to aid solubilization, filtered through a 0.22 μm syringe filter (Millipore), and stored the stocks in aliquots at −20 °C. After thawing an aliquot, we kept the stocks at 4 °C.

The cationic lipids DOTAP and 1,2-di-O-octadecenyl-3-trimethylammonium propane (DOTMA) were from Avanti Research, and the neutral helper lipid 1,2-Dioleoyl-sn-glycero-3-phosphoethanolamine (DOPE) from Polysciences. We solubilized the lipids in chloroform to reach 10 mg/ml concentration by either adding 100% chloroform directly into the vial, or by weighing the desired amount of the lipid into a 4 ml brown glass vial (Supelco) followed by addition of 100% chloroform to yield 10 mg/ml. We stored the chloroform stocks at −80 °C until use. To generate DOPE:DOTAP and DOPE:DOTMA (2:1, 1:1, and 0.5:1 ratios for both), we first added the desired amount of 10 mg/ml DOPE stock and then the required amount of DOTAP or DOTMA stock. In detail, to produce 1 ml of 2:1 1 mg/ml reagent stocks, we pipetted in a fume hood into 4 ml brown glass vials (Supelco) 66.5 μl of DOPE (10 mg/ml in chloroform) and 33.5 μl of DOTAP or DOTMA, for 1:1 ratio the respective volumes were 50 μl and 50 μl, and for 0.5:1 ratio 33.5 μl and 66.5 μl. After mixing the lipids well, we allowed the chloroform to evaporate from the vials, to aid evaporation we warmed the tubes by keeping them in hand and turning the tube around the vertical axis in ∼45 to 60 degree angle to increase the surface area and to aid lipid film formation. After evaporation of all chloroform, we resolubilized the lipids with 80% ethanol (20% of Milli-Q water plus 80% of absolute EtOH [≥99.5%]) by pipetting the solution up-down using a 1-mL Finnpipette F2 (Thermo Fisher Scientific) and vortexing, and stored the generated reagents in the glass vials at −20 °C until use. The DOPE:DOTAP and DOPE:DOTMA reagents were brought to RT prior to use and placed back at −20 °C until the next use.

### Transfection of cultured cells

The transfection reagents, except for Fugene HD, were first diluted in Opti-MEM (Gibco, cat no. 31985-047) to the desired working concentrations. Simultaneously, mCherry pDNA or mRNA stock was diluted in Opti-MEM to concentration 10 ng/μl. The diluted transfection reagents and the nucleic acids were subsequently combined at the required reagent-to-nucleic acid ratios: Lipofectamine 2000 (2:1, 4:1 and 6:1), PEI 25k and PEI 40k (1:1, 2:1, 3:1, 4:1, 5:1, 6:1, 7:1, 8:1, and 9:1), DOPE:DOTAP and DOPE:DOTMA combinations (1:1, 3:1, 6:1, 9:1, 12:1, and 15:1). In detail, for example, to generate 1:1 DNA to transfection reagent mix, we took 50 μl of plasmid mix (10 ng/μl in Opti-MEM) and mixed with 50 μl of the diluted (0.5 μl of transfection reagent plus 49.5 50 μl of Opti-MEM) transfection reagent. Fugene HD (1.5:1, 3:1 and 4.5:1) was added directly to the diluted nucleic acid, as described in the manufacturer’s protocol (as an example, 0.75 μl of Fugene HD would be mixed with 50 μl of plasmid mix [10 ng/μl in Opti-MEM] to yield 1.5:1 ratio). The resulting mixtures were pipetted up-down a few (3–5 times), vortexed briefly, spun down, and the complexes allowed to form by 15 min incubation at room temperature (RT). The resulting complexes were dispensed at 10 μl (5 μl of the Fugene HD transfection complexes) per well onto ViewPlate-96 Black, Optically Clear Bottom, Tissue Culture Treated (Revvity Health Sciences Inc., 6005182) plates, yielding 50 ng of nucleic acid to each well, and each condition represented by quadruplicates. Before transfection, the cells were detached using 0.25% trypsin-EDTA (Gibco, 25200056) and seeded on top of the transfection complexes at the density and in the culture medium detailed in Table 1. After replacing the media with fresh fully supplemented media 12 to 16 h postseeding/transfection, the cells were monitored daily, and the cells were fixed at 48 h after mCherry mRNA transfection and 72 h post mCherry pDNA transfection with 4% paraformaldehyde in PBS for 20 min at RT. The plates with fixed cells were stored at 4 °C until subsequent steps.

### Imaging of transfected cells and quantification of transfection rate

The nuclei of the fixed cells were stained by incubating with 50 μl/well of 0.2 μg/ml Hoechst 33342 (Thermo Fisher Scientific, Cat. no. 62249) in PBS 1 h at RT. Following staining, the buffer was replaced with 100 μl of PBS, and the plates were sealed with BackSeal (Revvity, Part number: 6005189) and stored at 4 °C until imaging. Automated fluorescence imaging was conducted with a Molecular Devices ImageXpress Nano high-content epifluorescence microscope equipped with a 10x objective lens and a 4.7-megapixel complementary metal oxide semiconductor camera (pixel size: 0.332 μm), a service provided by FIMM-HCA (Institute for Molecular Medicine Finland High content imaging and analysis unit, HiLIFE, University of Helsinki). Image analyses were done using CellProfiler-4 software (http://www.cellprofiler.org), with automated nuclei detection *via* the built-in Otsu algorithm. To identify the number of transfected cells, a five-pixel expansion around each nucleus was used to calculate the mean fluorescence intensity of the mCherry signal within each cell. An intensity threshold was applied to ensure that less than 0.01% of positive cells were detected in non-transfected wells. On average, over 30,000 cells per sample were analyzed, and each condition was represented by four wells. As an indicator of transfection efficiency, the percentage of mCherry-positive cells, relative to the total number of detected nuclei, was then quantified and plotted using Microsoft Excel (https://www.microsoft.com/en-us/microsoft-365/excel) and GraphPad (https://www.graphpad.com/).

### Gel retardation assay

To study the nucleic acid complex formation by the “in-house” transfection reagents, we mixed PEI 25k and PEI 40k at ratios of 1:1, 2:1, 3:1, 4:1, 5:1, 6:1, 7:1, 8:1, and 9:1 and DOPE:DOTAP and DOPE:DOTMA at ratios of 1:1, 3:1, 6:1, 9:1, 12:1, and 15:1 with pDNA. pDNA and the transfection reagents were diluted to a final volume of 25 μl in Opti-MEM (Gibco, cat no. 31985-047) and vortexed. The mixtures were incubated for 15 min at RT, and 5 μl (50 ng/well) of the mixture was loaded onto a 1% (w/v) agarose gel with GelRed nucleic acid stain (Biotium, cat. no. 41003-1). Electrophoresis was carried out in a 1X Tris-acetate-EDTA running buffer for 30 min at 90 V. DNA visualization was done using Gel Doc 2000 (Bio-Rad Laboratories).

### Cell viability assay

The cytotoxicity of the reagents for each of the 14 cell lines (HEK-293T, AV3, BHK-21, Caco2, Caki-1, Calu-1, CHO, Huh-7, Vero-E6, XTC, JAR, BVK-168, SK-N-SH, and I/1Ki) was evaluated using the CellTiter-Glo 2.0 cell viability assay (Promega, Cat. no. G9242). Only the highest concentration ratio of each reagent—Lipofectamine 2000 (6:1), Fugene HD (4,5:1), PEI 25K (9:1), PEI 40K (9:1), DOPE:DOTAP (15:1), and DOPE:DOTMA (15:1)—was tested for cell viability. The transfection complexes and cells were prepared similarly as before and plated on ViewPlate-96 Black, Optically Clear Bottom, Tissue Culture Treated (Revvity Health Sciences Inc., 6005182) plates with four replicates of each combination. The media was changed after 16 h, and cytotoxicity was measured 48 h posttransfection. 100 μl of CellTiter-Glo 2.0 reagent was added into each well and the plates were mixed on an orbital shaker for 2 min. The plates were then incubated for 10 min at RT. Luminescence was measured (1 s/well) with HIDEX Sense Microplate reader. The cytotoxicity of the reagents was compared to cells grown under the same conditions without DNA and transfection reagent addition.

## Data availability

All of the experiment and measurement data are contained within the article. Raw data are available upon request, contact emilia.timin@helsinki.fi or jussi.hepojoki@helsinki.fi.

## Conflict of interest

The authors declare that they have no conflicts of interest with the contents of this article.
